# The effect of oral preexposure prophylaxis on the progression of HIV-1 seroconversion

**DOI:** 10.1097/QAD.0000000000001577

**Published:** 2017-08-31

**Authors:** Deborah Donnell, Eric Ramos, Connie Celum, Jared Baeten, Joan Dragavon, Jordan Tappero, Jairam R. Lingappa, Allan Ronald, Kenneth Fife, Robert W. Coombs

**Affiliations:** a Vaccine and Infectious Disease Division, Fred Hutchinson Cancer Research Center; b Department of Laboratory Medicine; c Department of Global Health; d Department of Epidemiology; e Department of Medicine; f Department of Pediatrics, University of Washington, Seattle, Washington; g Division of Global Health Protection, Center for Global Health, CDC, Atlanta, Georgia, USA; h Departments of Medical Microbiology and Internal Medicine, University of Manitoba, Winnipeg, Manitoba, Canada; i Department of Microbiology and immunology, Indiana University, Indianapolis, Indiana, USA.

**Keywords:** early HIV-1 seroconversion, Fiebig stages, HIV-1 RNA, HIV-1 testing, preexposure prophylaxis

## Abstract

**Objective:**

To investigate whether oral preexposure prophylaxis (PrEP) alters timing and patterns of seroconversion when PrEP use continues after HIV-1 infection.

**Design:**

Retrospective testing of the timing of Fiebig stage HIV-1 seroconversion in the Partners PrEP Study, a randomized placebo-controlled clinical trial of PrEP conducted in Kenya and Uganda.

**Methods:**

Specimens from 138 seroconverters were collected every 3 months and when HIV-1 infection was suspected based on monthly rapid HIV-1 tests. Progression of seroconversion was compared between randomized groups (PrEP versus placebo) and per-protocol groups (placebo versus PrEP participants with detectable tenofovir during the seroconversion period) using laboratory assessment of Fiebig stage. Delay in site-detection of seroconversion and association with PrEP drug-regimen resistant virus were assessed using logistic regression. Analysis of time to each Fiebig stage used maximum likelihood estimation with a parametric model to accommodate the varying lengths of HIV-infection intervals.

**Results:**

There was a significant increase in delayed site detection of infection associated with PrEP (odds ratio = 3.49, *P* = 0.044). Delay in detection was not associated with increased risk of resistance in the PrEP arm (odds ratio = 0.93, *P* = 0.95). Estimated time to each Fiebig stage was elongated in seroconverters with evidence of ongoing PrEP use, significantly for only Stage 5 (28 versus 17 days, *P* = 0.05). Adjusted for Fiebig stage, viral RNA was ∼2/3 log lower in those assigned to PrEP compared with placebo; no differences were found in Architect signal to cut-off at any stage.

**Conclusion:**

Ongoing PrEP use in seroconverters may delay detection of infection and elongate seroconversion, although the delay does not increase risk of resistance.

## Abstract 2

Supplemental Digital Content is available in the text

## Introduction

Multiple randomized clinical trials have shown that with good adherence, preexposure prophylaxis (PrEP), substantially reduces risk of HIV-1 acquisition [1–6]. This prevention strategy requires frequent high-quality HIV-1 testing among PrEP users to detect acute/early HIV-1 infection and minimize risk of resistance. Nonadherence to PrEP provides little HIV-1 protection but at the same time little risk of resistance if the patient is infected [7], whereas high adherence to PrEP blocks most transmissions [8]. For those who acquire HIV-1 in spite of PrEP – whether from sporadic adherence, or potentially a breakthrough with high adherence – it is unknown if PrEP use modifies the progression of seroconversion or the natural evolution of HIV-1 biomarkers.

Fiebig *et al.*[9] developed a classification schema of primary HIV-1 infection using sequential assay reactivity to identify six distinct laboratory stages of acute/early HIV-1 infection over approximately a 3-month period following HIV-1 acquisition. The Fiebig stages document the progression of infection from an initial ‘eclipse phase’ in local mucosal tissue, through dissemination to regional lymph nodes to systemic spread accompanied by high levels of HIV-1 replication in the blood [10,11].

The primate model of PrEP has shown reduced peak virus load and suppressed maturation of antibody avidity with PrEP break-through of SHIV_SF162P3_ infection but little impact on the timing of seroconversion and neutralizing or binding antibody levels [12]. If PrEP is used during all or part of acute HIV-1 infection, when antibody response develops, it is biologically plausible that these biomarkers of acute HIV-1 infection, HIV-1 RNA and p24 antigen and antibodies against HIV-1 will be delayed, attenuated or perhaps even skipped. Within a placebo-controlled trial of PrEP, we assessed whether PrEP use affected the detection of infection, timing of Fiebig stages or virological and immunological response to HIV-1 infection.

## Methods

### Study population

Participants in the double-blinded Partners PrEP Study were enrolled in Kenya and Uganda, and randomized 1 : 1 : 1 to receive daily emtricitabine/tenofovir (FTC/TDF), TDF or placebo. Participants were seen monthly for HIV-1 testing and provision of a 1-month supply of study medication [13]. Those with reactive or discordant rapid tests were considered possible seroconverters and confirmed by third-generation enzyme immune assays (EIA) at the local laboratory. Study drug was temporarily withheld for any HIV-reactive test and permanently discontinued once seroconversion was confirmed. Plasma and serum samples were stored at months one, three and each subsequent quarterly visit, at any visit with a reactive HIV-1 test, and for seroconverters, at visits within a month and then quarterly thereafter. HIV-1 infections were confirmed centrally from stored samples.

Participants (*N* = 4747) were enrolled between July 2008 and November 2010. In July 2011, the independent data monitoring committee (DMC) recommended that use of placebo be discontinued because the PrEP intervention had demonstrated overwhelming efficacy. The study continued participants in the active arms and placebo-arm participants were unblinded and offered rerandomization to the continuing active arms. Thus, there were two study periods: a primary randomized period with participants assigned 1 : 1 : 1 to placebo, TDF and FTC/TDF and the post-DMC placebo unblinding period in which unblinded placebo participants were rerandomized 1 : 1 to TDF and FTC/TDF [8,13]. All participants provided written informed consent in English or their local language, including reconsent for rerandomization (ClinicalTrials.gov number NCT00557245). The study protocol was approved by the University of Washington Human Subjects Review Committee and ethics review committees at each study site.

### Laboratory methods

Sites used the Determine (Alere, Orlando, Florida, USA) rapid test kit run in parallel with any of the Unigold (Trinity Biotech, Wicklow, Ireland), Bioline (Standard Diagnostics, Yongin, Korea) or STAT-PAK (Chembio Diagnostic Systems, Medford, New York, USA) whole blood rapid tests; reactive rapid tests were confirmed with either a third or fourth-generation confirmatory EIA serum test [14,15]. Additional testing, performed at the Department of Laboratory Medicine, University of Washington (Clinical Laboratory Improvement Amendment certified and College of American Pathologists accredited) established the last HIV-1 nonreactive visits and assessed Fiebig stage using plasma. Laboratory testing included: HIV-1 RNA detection using the Abbott m2000rt Real Time HIV-1 RNA (Abbott Molecular, Chicago, Illinois, USA) with limit of detection of 40 copies/ml; HIV-1 p24 antigen/HIV-1/2 antibody detection using the ARCHITECT HIV-1/2 Ag/Ab Combo CMI assay (Abbott Diagnostics) and Bio-Rad HIV-1/2 Ag/Ab Combo EI assay; IgM/IgG antibody detection, confirmation and discrimination using the Multispot HIV-1/HIV-2 rapid test (Bio-Rad Laboratories, Redmond, Washington, USA); and western blot [Genetic Systems HIV-1 WB assay (Bio-Rad Laboratories)]. A Multispot rapid test was considered positive if both HIV-1 dots developed, per the manufacturer; a single HIV-1 spot was considered indeterminate. The western blot (WB) was considered HIV-1 positive if any two of the p24, gp41 or gp120/160 were reactive: any other blot reactivity was considered indeterminate.

Plasma TDF concentrations were determined in selected archived plasma samples by previously described ultra-performance liquid chromatography–mass spectrometry assay methods [16,17]. Calibration standards ranged from 0.31 to 1280 ng/ml. Drug susceptibility genotype was performed and reported elsewhere for all the PrEP study HIV-1 infections, including the discordant HIV-1-infected partners [7,8].

### Outcomes

To investigate the effect of PrEP on HIV-1 during acute/early infection, we assessed: time to site detection of HIV-1 infection, time to each Fiebig stage, HIV-1 viral RNA and overall antibody response [as measured by Architect signal to cut-off (S/CO) ratio]. In addition, we assessed whether occurrence of resistant virus was associated with delay in site detection of infection.

Time to site detection of HIV-1 infection was from time of sample with first evidence of infection to time of site-detected seroconversion. Analysis of time to Fiebig stage was based on all samples available during the ‘seroconversion period’, defined as from the last HIV-1-uninfected visit to when Fiebig stage six was first reached. Although HIV-1 testing was monthly, samples were stored every 12 weeks. Delay in site detection of seroconversion was defined as more than 100 days between first HIV-1-infected sample and site detection of infection, to allow for the maximum interval between stored samples. Similarly, as the probability of detecting early Fiebig stages is higher with shorter sampling intervals, the length of time between samples was explicitly incorporated into the analysis estimating Fiebig stage duration (see Supplementary Appendix 1). Both analyses exclude seroconverters who missed study visits and did not receive HIV-1 testing at the site for more than 100 days, as they do not contribute information about early progression of seroconversion.

Treatment arm was defined as PrEP (TDF/FTC or TDF) if randomized to PrEP at any time during the seroconversion period (defined above). If site rapid tests were nonreactive, participants were randomized and started on PrEP. Seroconverters whose infection was not detected prior to starting PrEP were included in the PrEP group. Analyses assessed exposure to PrEP both ‘as-randomized’ and ‘as-treated’, with the latter defined as detectable TDF concentrations in plasma in any sample during the seroconversion period. Detectable TDF concentrations at the last HIV-1-uninfected visit were excluded if the participant subsequently missed study visits for more than 100 days.

The testing algorithm used to define the last uninfected visit and Fiebig stage definitions are shown in Table S1. We note a modification of the original Fiebig staging in the definition of Stage 3, substituting the Multispot rapid test for Fiebig's older ‘sensitive’ EIA; a positive Multispot has been characterized as occurring 7 days prior to a positive WB [18]. Because of the potential for PrEP to suppress HIV-1 RNA level in plasma, detectable HIV-1 RNA was not required for Fiebig stages two to six.

### Statistical methods

Delay in detection associated with PrEP was assessed using logistic regression, as was occurrence of drug resistant virus and delayed detection in the PrEP arm.

A parametric model was used to test whether cumulative time to reach each Fiebig stage was attenuated by PrEP use. Each participant's sequence of available data consisted of time of last HIV-1 uninfected sample (*t*_0_ = 0); time and stage of first HIV-infected sample (*t*_1_), and a series of subsequent times and stage of infection up to the first Fiebig stage 6 sample (*t*_*n*_). The ‘infection interval’ was defined as the time between the last HIV-1-uninfected and first HIV-1-infected sample (0,*t*_max_ = *t*_1_). Time to each Fiebig stage, *T*_*k*_, after (unobserved) time of infection was assumed to follow an Exponential waiting time distribution with mean 1/*λ*_*k*_. The assumption of an exponential waiting time was judged appropriate as the estimated durations in the placebo arm closely match those originally reported by Fiebig *et al.*[9]. The time of infection was assumed to be uniformly distributed in the infection interval (0,*t*_max_). The parametric survival distribution and contributions to the likelihood for time to Stage *k* under these assumptions are given in Appendix 1 (Supplementary material).

To test the hypothesis that the time to reach Fiebig stage k was longer for persons on PrEP than placebo, we modelled  
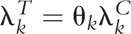
, where  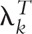 is the event rate for PrEP arm,  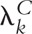 the event rate for placebo arm and θ_*k*_ the relative increase in time to Stage *k* attributed to PrEP use. Note that time to Stage 1 was inestimable, as the earliest possible detection of infection is synonymous with Stage 1.  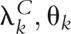 were estimated using maximum likelihood; 95% confidence interval (CI) were computed using Fisher Information. *P* values were computed via bootstrap estimation, using permutations of the assignment of PrEP versus placebo.

Comparison of viral load (log_10_ RNA copies/ml) and Architect S/CO between groups used Generalized Estimating Equation models adjusted for Fiebig stage.

## Results

There were a total of 138 HIV-1 seroconverters: 67 were assigned to PrEP during the seroconversion period (40 TDF, 27 FTC/TDF), and 71 received placebo. Fifteen were HIV-1 infected (HIV-1 RNA detected) but seronegative at initial randomization, 111 became infected on study, nine infections occurred during an off-study drug period and three were infected at placebo rerandomization. About half were men (46%), the median age was 30 and median viral load of their HIV-1-infected partners was more than 20 000 copies per/ml (Table 1). Among the 67 randomized to PrEP, 64 were assessed for TDF in plasma during the seroconversion period, and 31 (48%) had detectable TDF during that period, of whom15 had TDF concentrations more than 40 ng/ml, consistent with daily dosing [19,20].

**Table 1:** Characteristics of seroconverters, timing of site detection of infection and viral load during seroconversion (*N* = 138).

	PrEP, *N* = 67	Placebo, *N* = 71
Male	27 (40%)	37 (52%)
Plasma HIV-1 RNA viral load of partner (median log_10_ copies/ml)	4.33	4.43
Age (median)	31	30
Infected at randomization	9 (14%)	6 (8%)
Time to detect seroconversion at site^a^	*N* = 58	*N* = 71
0 days	21 (36%)	36 (51%)
Within 100 days	27 (47%)	31 (44%)
>100 days	10 (17%)	4 (6%)
Plasma HIV-1 RNA viral load for samples in each Fiebig stage				
	PrEP		Placebo	
	Undetectable	Mean log_10_ VLb	Undetectable	Mean log_10_ VL
Overall	13/121 (11%)		4/134 (3%)	
Stage 2	2/7 (29%)	4.49	0/10 (0%)	5.98
Stage 3	0/2 (0%)	4.12	0/1 (0%)	5.54
Stage 4	1/10 (10%)	3.71	2/11 (18%)	4.70
Stage 5	3/38 (8%)	4.07	1/43 (2%)	4.76
Stage 6	7/64 (11%)	4.13	1/69 (1%)	4.62

PrEP, preexposure prophylaxis; VL, viral load.

^a^Nine seroconverters in the PrEP arm and 0 on the placebo arm had no site HIV-1 test for more than 100 days prior to first HIV-1 infected visit. These participants are not included in the assessment of time from first HIV-1 infected sample to site detection of seroconversion.

^b^Samples with undetectable viral load were assigned 40 copies/ml when computing the mean.

### Detection of HIV-1 seroconversion

**Fig. 1:**
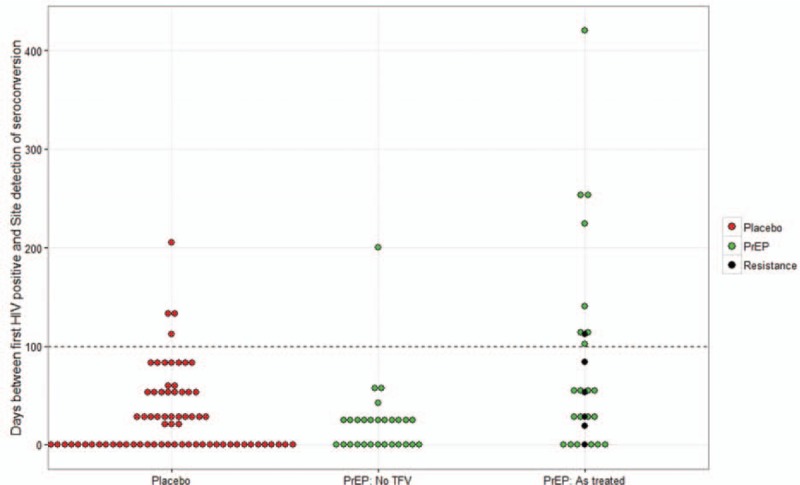
Time between first HIV-infected sample and site detection of seroconversion (*N* = 129).

Assessment of delay in detection of infection included 129 seroconverters; nine with no site HIV-1 test in the 100 days prior to detection of seroconversion because of missed study visits were excluded (Table 1; Fig. 1). For 57 (44%), the first infected visit (identified by subsequent central lab testing) coincided with site detection of seroconversion; for a further 58 (45%), site diagnosis of seroconversion occurred within 100 days of the first infected visit. Of the 14 for whom infection was not detected by monthly site HIV-1 testing for more than 100 days, four were assigned to placebo and 10 to PrEP, nine of these were in the as-treated group. The odds ratio (OR) for a delay more than 100 days in detection of seroconversion for PrEP versus placebo was 3.49 (95% CI 1.03–11.8, *P* = 0.044); for PrEP as-treated versus placebo, OR = 7.18, (95% CI 2.00–25.7, *P* = 0.002). As previously reported [7,13], six PrEP seroconverters had virus with mutations associated with resistance to TDF or FTC/TDF; for five of six where the partner was the source of the virus, resistance appears to have been selected by PrEP. All of these were in the as-treated group, but there was no association in the PrEP arm between having a resistant mutation and delay in site detection of infection (OR = 0.925, *P* = 0.95; Fig. 1).

Each dot represents one seroconverter. Time of 0 days implies site detected seroconversion occurred at the first HIV-infected visit. The preexposure prophylaxis arm is displayed as two groups: preexposure prophylaxis as-treated, participants who had tenofovir detected during the seroconversion interval; and preexposure prophylaxis with no tenofovir detected. Black filled dots indicate the participants who had resistance mutations at seroconversion.

### Progression and time to reach Fiebig stages

**Fig. 2:**
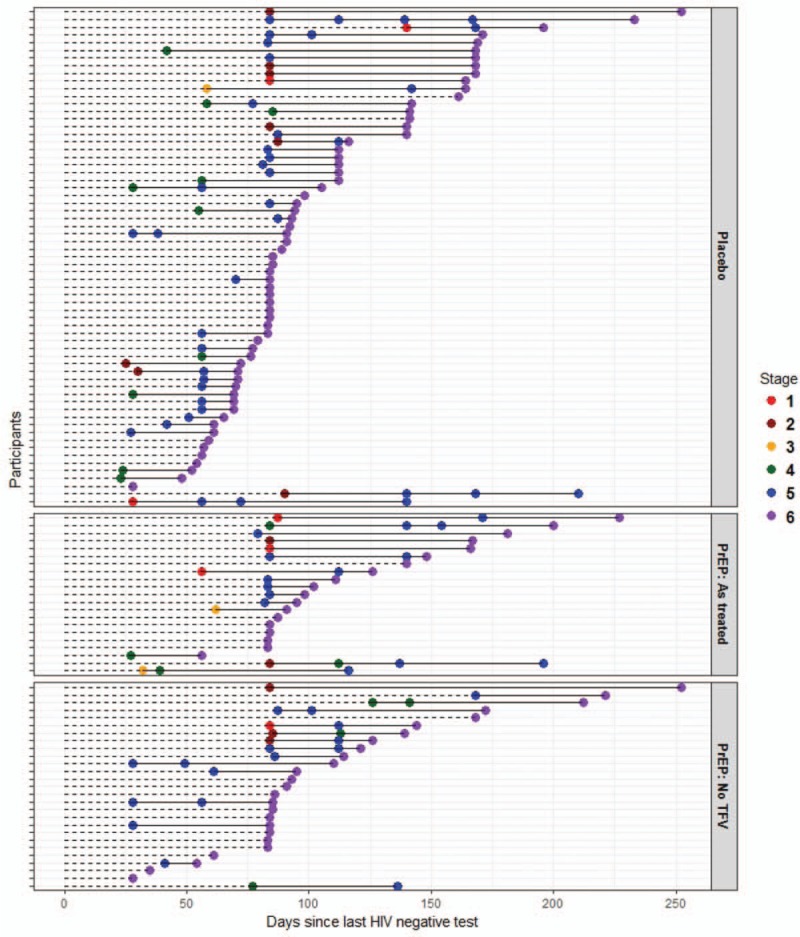
Fiebig stage observed in placebo, preexposure prophylaxis as-treated and preexposure prophylaxis with no tenofovir detected groups.

All 138 seroconverters were observed though to Fiebig Stage 5 or 6 (i.e. final sample was western blot positive), with none initiating antiretroviral therapy for treatment during the seroconversion period. A total of 88 (64%) seroconverters had samples from more than one Fiebig stage; the remaining 50 (36%) were Stage 6 at their first HIV-infected visit. Of the 138 seroconverters, 113 were included in the analysis of time to Fiebig stage: 25 were excluded, 16 because they had no HIV-1-uninfected sample and nine, because they had more than 100 days with no site HIV-1 test prior to detection of seroconversion. Figure 2 shows the infection interval and the stages detected during seroconversion period by time since beginning of infection interval. Amongst these 113, 74 (65%) seroconverters had samples from more than one Fiebig stage; and 39 (35%) were Stage 6 at their first HIV-infected visit. Randomization to PrEP was not associated with a statistically significant increase in time to Fiebig stage, for any stage (Table 2). However, comparing as-treated PrEP with placebo groups, a statistically significant relative increase in time to reach stage 5 was observed (θ_5_ = 0.599, *P* = 0.05), corresponding to an increase in mean days to full western blot from 49 days amongst placebo to 80 days for seroconverters taking PrEP. There was a consistent pattern of relative increase in time to reach each Fiebig stage in PrEP compared with placebo at all stages in both as-randomized and as-treated comparisons, and consistently higher relative increases in as-treated compared with as-randomized comparisons against placebo.

The tested samples for each participant is displayed on a single line. Sample times are shown at days since last HIV-1 uninfected visit, and stages for each sample displayed by colour. Time interval of infection (last HIV-1 uninfected to first infected sample) is shown by dashed line. Time interval for acute/early seroconversions (first infected sample to Fiebig stage 6) is shown by a solid line.

**Table 2:** Time to reach Fiebig stage.

	Estimated mean number of days to reach Fiebig stage^a^		Relative rate to reach stage for PrEP versus placebo^b^ 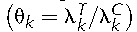	*P* value^c^
	PrEP	Placebo		
PrEP: as randomized	*N* = 48	*N* = 65		
Stage 2	5	3	0.503	0.288
Stage 3	11	9	0.818	0.621
Stage 4	13	10	0.781	0.479
Stage 5	22	17	0.764	0.285
Stage 6	60	49	0.820	0.490
PrEP: as treated	*N* = 21	*N* = 65		
Stage 2	10	3	0.264	0.078
Stage 3	16	9	0.578	0.255
Stage 4	19	10	0.524	0.132
Stage 5	28	17	0.599	0.053
Stage 6	80	49	0.612	0.197

PrEP, preexposure prophylaxis.

^a^Calculation of mean number of days to 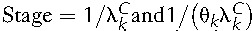 respectively.

^b^Rates estimated by maximum likelihood assuming uniform distribution of (unobserved) infection time and exponential waiting time to each Fiebig stage.

^c^*P* values based on bootstrap permutation test.

### Plasma viral load during seroconversion in preexposure prophylaxis versus placebo participants

Plasma HIV-1 RNA level, adjusted for Fiebig stage of sample, was ∼2/3 log_10_ lower in those assigned to PrEP compared with placebo (−0.64 log_10_ copies/ml; 95% CI −0.94 to −0.34; *P* < 0.001) and ∼3/4 log lower in PrEP as-treated compared with placebo (−0.74 log_10_ copies/ml; 95% CI −1.11 to −0.36; *P* < 0.001). For samples in Stages 2–6 (Table 1), four of 134 (3%) on placebo and 13 of 121 (11%) on PrEP had undetectable viral load (OR = 3.9; 95% CI 1.24–12.4; *P* = 0.02). To exclude an integrase target detection problem with the Abbott m2000 HIV-1 RNA test, these samples were also confirmed to be HIV-1 RNA negative using the Roche COBAS AmpliPrep/COBAS TaqMan HIV-1 test, v2.0 (Roche, Branchburg, New Jersey, USA), which targets HIV-1 LTR and gag.

**Fig. 3:**
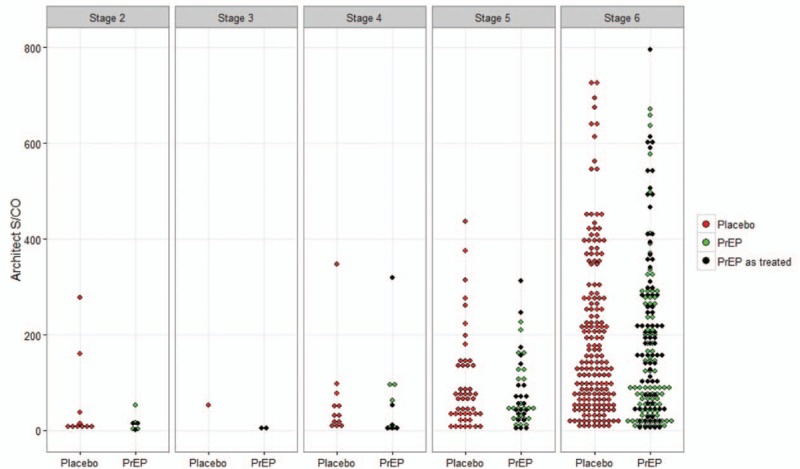
Architect signal to cut-off ratio is plotted for each sample by stage and arm.

No differences were found in Architect S/CO comparing PrEP with placebo in as-randomized or as-treated comparisons (Fig. 3).

Each dot represents a sample tested from the seroconverter interval (between the last HIV-1-uninfected sample and first Fiebig stage 6 sample). The filled black dot indicates a sample from a participant in the preexposure prophylaxis arm who had detectable tenofovir during their seroconversion interval. No differences in signal to cut-off ratio were observed between groups.

## Discussion

In this analysis of a randomized, placebo controlled trial of PrEP, we showed that PrEP delayed the time to detect seroconversion for those participants who continued to take PrEP during acute/early clade C HIV-1 infection. Nonetheless, the majority of HIV-1 infections were detected within 3 months, which corresponds to the currently recommended HIV-1 testing frequency for patients on PrEP. We also observed a consistent trend of increased time for Fiebig stage progression among the seroconverters with TDF-monitored evidence of continued PrEP exposure. A statistically significant delay occurred only for Fiebig stage 5, likely because it has the longest duration and thus was observed most frequently. Although our analysis suggests PrEP may elongate seroconversion, the delay in detection was not associated with developing resistant virus, thus the clinical consequences on seroconversion appear unlikely to be significant.

Our findings are similar to those from a randomized trial of TDF PrEP among IDUs, which also reported a substantial delay in detection of clade A/E HIV-1 seroconversion in the TDF arm using the OraQuick oral fluid test [21]. A study among women in South Africa also observed a delay in antibody maturation following clade C HIV-seroconversion in women assigned to TFV gel [12]. In primate studies, delay in seroconversion was not observed in PrEP breakthrough infections, although maturation of antibody avidity was delayed [22]. We did not assess antibody maturation in our study.

Consistent with the primate studies [22,23], we found that PrEP suppressed viral replication during seroconversion, a reassuring consequence of antiretroviral exposure during acute and early infection. Lower viral loads have not been observed in other PrEP trials [2,24,25], although this may be attributable to lower adherence to PrEP. In two recently reported cases of multidrug-resistant breakthrough infections in which self-report and drug detection indicated high adherence to PrEP, viral load remained suppressed or low-throughout seroconversion [26,27].

Naturally occurring (i.e. placebo arm) suppression of viral load during acute/early infection was also observed, as was prolonged time to detect seroconversion, suggesting that there are also host determinants of the seroconversion process. To date, studies of acute and early HIV-1 seroconversion have used detection of viral RNA to define the earliest evidence of infection immediately following the eclipse stage [28–30]; thus, naturally occurring suppression of RNA viral load during acute/early infection is not often observed, although it has been previously reported [31,32]. Elongation of time to develop detectable HIV-1 infection beyond 4 months could reflect either the performance of the HIV-1 rapid tests [33–35] or a delay in development of HIV-1 antibody [36,37].

Prolongation of time to full seroconversion could indicate alteration of the early immunological response to HIV-1 or delayed serologic progression in response to lower viral replication. The lack of difference in the Architect S/CO ratios indicated no evidence of differences in the overall antibody response to HIV-1 infection; thus, a delay in progression to Fiebig stage 5 is most likely attributable to lower viral burden during seroconversion.

Resistance mutations to TDF or FTC have been rare in PrEP clinical trials: eight (18%) HIV-1 infections with mutations occurred among 44 individuals HIV-1 infected at enrolment (two on placebo and six on PrEP); among incident HIV-1 infections, drug-resistant infections have been detected in one of 254 on placebo and five of 164 on PrEP [38]. Recently, two cases of multidrug-resistant breakthrough infections have been reported in patients with consistent adherence to daily PrEP [26,27]. It is plausible that drug-resistant mutations may be more likely to develop if PrEP significantly delays diagnosis of HIV-1 infection. In our study, although all resistance mutations occurred in those who continued PrEP after infection, mutations conferring resistance to FTC/TDF were not related to delayed detection. It seems likely that drug exposure was low when infection occurred, and ongoing selective pressure, because of either high or no adherence, was not sufficient for resistance to be common. Reassuringly, there is little indication that a delay in detection of infection increases the chance of resistance mutations.

At the time of this study, antibody-based rapid tests were in use as the standard for detection of new HIV-1 infections at participating sites. In just over half the cases, these tests did not detect HIV-1 infection at the first HIV-1-infection visit, as these rapid tests are not highly sensitive during early infection. Similar findings of imperfect detection of early infection with rapid EIA tests have been reported in other PrEP studies [34,35]. The more sensitive EIA and chemiluminescent microparticle immunoassay Ag/Ab tests now available, and recommended for use in patients on PrEP, would likely detect infections at the earlier Fiebig stages as we demonstrated. The higher frequency of visits with undetectable HIV-1 RNA during seroconversion among those assigned to PrEP suggests that use of viral RNA as a confirmatory or diagnostic test may not be adequate, and total nucleic tests for cell-associated HIV-1 RNA and DNA may be required to rule out HIV-1 infection in the presence of inconclusive HIV-1 diagnostic tests.

A significant limitation in our study of Fiebig stages is the 1–3-month gaps between stored samples needed for staging, compared with the 1–2-week durations for Fiebig stages 1–4. With the limited number of seroconverters on PrEP, we had limited power to detect changes in time to reach each stage. The strengths of the study are that in this cohort, with high retention and relatively high adherence to both visits and PrEP, samples were stored and available every 3 months for almost every seroconverter during seroconversion. Our testing strategy did not duplicate the earlier but elegant Fiebig staging schema because of the change in HIV-1 diagnostic platforms from second and third-generation assays used by Fiebig to third and fourth-generation assay platforms used in our study; as such, our staging approach for acute/early HIV-1 infection should be considered a modification to the original Fiebig staging. Nevertheless, the close similarity in stage duration between the original and modified schemas suggests that current-testing algorithms can be used to define a contemporary Fiebig staging schema for acute/early HIV-1 infection [39].

### Conclusion

The 2015 WHO recommendation that PrEP be implemented as part of an effective prevention package for persons at substantial risk of HIV-1 infection is leading to increasing scale-up of PrEP. Delay in detection of HIV-1-infection as a result of PrEP use would be a concern if the recommended quarterly HIV testing missed diagnoses and inadvertently prolonged PrEP exposure after infection, thereby increasing risk of resistance mutations; our study is reassuring in not finding evidence of this risk. Our study suggests that delay in progression of seroconversion is likely a result of PrEP's suppression of viral replication: as aligned with the goal of early treatment interventions, this may ultimately prove to be beneficial to the patient. Future study of delay or even aborted development of viral and antibody markers in HIV-1 seroconverters with continued PrEP exposure will be important in the United States and other settings where much more frequent testing is now routine. We concur with the need to use highly sensitive rapid HIV-1 tests in patients using PrEP, so that delays in developing full western blot pattern (or equivalent) will not delay detection of HIV-1 infection. The potential benefit for TDF-containing oral PrEP to prevent HIV-1 acquisition remains high, and our analysis adds support to a risk–benefit ratio clearly in favour of continuing the effort to scale-up PrEP in populations with substantial HIV-1 risk.

## Acknowledgements

D.D., C.C. and J.B. contributed to the design and execution of the study had full access to the data. D.D. conducted the statistical analyses and wrote the first draft of the article. E.R., J.D. and R.B. conducted the laboratory testing and contributed to the design of the study. J.T., J.R.L., A.R. and K.F. contributed to the design and execution of the study. All authors contributed to critical review and approved the final article.

We are grateful to the couples who participated in this study for their motivation and dedication. We thank the members of the independent Data and Safety Monitoring Board for their wisdom and guidance throughout the trial. We also thank James Bremer PhD and Cheryl Jennings, NIH/NIAID/DAIDS Virology Quality Assurance Program, Rush Medical College, Chicago, Illinois, USA for the confirmatory Roche TaqMan testing (HHSN272201200023C and HHSN266200500044C).

The Partners PrEP Study was funded through a research grant from the Bill & Melinda Gates Foundation (grant ID no. 47674). Central laboratory support for HIV-1 testing was provided in part through the University of Washington Center for AIDS Research, funded by the US National Institutes of Health under award number P30 AI027757 and UM1-AI106701. Gilead Sciences donated the study medication but had no role in data collection or analysis.

Disclaimers: The findings and conclusions in this article are those of the authors and do not necessarily represent the views of the Centers for Disease Control and Prevention.

We also acknowledge the tireless efforts of the study team.

Partners PrEP Study Team: *University of Washington Coordinating Center and Central Laboratories, Seattle, USA*: Connie Celum (principal investigator, protocol co-chair), Jared M. Baeten (medical director, protocol co-chair), Deborah Donnell (protocol statistician), Robert W. Coombs, Lisa Frenkel, Craig W. Hendrix, Jairam R. Lingappa, M. Juliana McElrath.

*Study sites and site principal investigators*: Eldoret, Kenya (Moi University, Indiana University): Kenneth H. Fife, Edwin Were; Kabwohe, Uganda (Kabwohe Clinical Research Center): Elioda Tumwesigye; Jinja, Uganda (Makerere University, University of Washington): Patrick Ndase, Elly Katabira; Kampala, Uganda (Makerere University): Elly Katabira, Allan Ronald; Kisumu, Kenya (Kenya Medical Research Institute, University of California San Francisco): Elizabeth Bukusi, Craig R. Cohen; Mbale, Uganda (The AIDS Support Organization, CDC-Uganda): Jonathan Wangisi, James D. Campbell, Jordan W. Tappero; Nairobi, Kenya (University of Nairobi, University of Washington): James Kiarie, Carey Farquhar, Grace John-Stewart; Thika, Kenya (University of Nairobi, University of Washington): Nelly R. Mugo; Tororo, Uganda (CDC-Uganda, The AIDS Support Organization): James D. Campbell, Jordan W. Tappero, Jonathan Wangisi.

Data management was provided by DF/Net Research, Inc. (Seattle, USA) and site laboratory oversight was provided by Contract Laboratory Services (CLS) of the Wits Health Consortium (University of the Witwatersrand, Johannesburg, South Africa).

### Conflicts of interest

There are no conflicts of interest.

Data presented previously at R4P Conference, Chicago, October 2016. Abstract OA03.01.

## Supplementary Material

Supplemental Digital Content

## Supplementary Material

Supplemental Digital Content
