# Supplementary material for: The effect of oral preexposure prophylaxis on the progression of HIV-1 seroconversion
Source: AIDS. 2017 Aug 31;31(14):2007–16. doi: 10.1097/QAD.0000000000001577 (PMC5578893; doi:10.1097/QAD.0000000000001577)
Supplement: Supplemental Digital Content [file aids-31-2007-s001.docx]

Appendix 1

The sequence of available data for Fiebig stages consists of time of last HIV-1 uninfected sample ${(t}_{\text{0}}=0)$; time and stage of first HIV-infected sample${(t}_{\text{1}})$, and a series of subsequent times and stage of infection up to the first Fiebig stage 6 sample ($t_{\text{n}})$. Each person’s “infection interval” is defined as the time between the last HIV-uninfected and first HIV-infected sample $({0, t}_{\text{max}}=t_{\text{1}})$.

Time to each Fiebig stage, $T_{k},$ after (unobserved) time of infection is assumed to follow an Exponential waiting time distribution with mean $1/{\lambda_{k}}$. The time of infection is assumed to be Uniformly distributed in the infection interval ($0, t_{\text{max}})$. The parametric survival distribution for the time to Stage k is:

$$P\left( T_{k}>t \right)= S\left( t \right)= 1-\frac{1}{t_{\text{max}}}\int_{x=0}^{t} \int_{{y=0}}^{t-x} {\lambda_{k}e}^{-\lambda_{k}y}dy dx$$

$$=\left\{ \begin{matrix} 1-\frac{1}{t_{\text{max}}}\left( t-\frac{1}{\lambda_{k}}\left( 1-e^{\lambda_{k}t} \right) \right)\text{when} t< t_{\text{max}} \\ \frac{1}{{\lambda_{k}t}_{\text{max}}}e^{\lambda_{k}(t-t_{\text{max}})}\text{ when} t \geq t_{\text{max}} \end{matrix} \right.$$

For each person, for Fiebig stage *k*, the contribution to the likelihood is

1. 1 - S(t_1_; $\lambda_{k}$) if the stage observed at t_1_ is greater than k
2. S(t_i-1_; $\lambda_{k}$) – S(t_i_; $\lambda_{k}$) if stage observed at prior time t_i-1_ is < k and at time t_i_ is > k
3. 0, otherwise.
