# Supplementary material for: The effect of oral preexposure prophylaxis on the progression of HIV-1 seroconversion
Source: AIDS. 2017 Aug 31;31(14):2007–16. doi: 10.1097/QAD.0000000000001577 (PMC5578893; doi:10.1097/QAD.0000000000001577)
Supplement: Supplemental Digital Content [file aids-31-2007-s002.docx]

Table S1: Laboratory testing used to define modified Fiebig stages

| **Stage of Infection** | **Modeled cumulative time to stage (mean, 95% CI)^[^**[**^9^**](#_ENREF_9)**^]^** | **Abbott m2000rt Real Time HIV-1 RNA** | **ARCHITECT HIV-1/2 Ag/Ab Combo CMI OR Bio-Rad HIV-1/2 Ag/Ab Combo** | **Multispot HIV-1/HIV-2 rapid** | **Genetic Systems HIV-1 WB assay** |
| --- | --- | --- | --- | --- | --- |
| HIV-`1 Uninfected |  | Undetectable | Negative | Negative | Negative |
| Stage 1 | 5 (3, 8) | **Detectable** | Negative | Negative | Negative |
| Stage 2 | 10.(7, 14) | Not used | **Positive** | Negative | Negative |
| Stage 3 | 14 (10, 17) | Not used | Positive | **Positive** | Negative |
| Stage 4 | 19 (15, 23) | Not used | Positive | Positive | **Indeterminate** |
| Stage 5 | 89 (47, 130) | Not used | Positive | Positive | **Positive – p31 band** |
| Stage 6 | Open ended | Not used | Positive | Positive | **Positive + p31 band** |
